# Low Prevalence of *Toxoplasma gondii* in Dogs From Central China

**DOI:** 10.3389/fcimb.2022.885348

**Published:** 2022-04-28

**Authors:** Niuping Zhu, Liulu Yang, Shilin Xin, Wei Huang, Yibao Jiang, Yurong Yang

**Affiliations:** ^1^ College of Veterinary Medicine, Henan Agricultural University, Zhengzhou, China; ^2^ College of Animal Science, Henan Agricultural University, Zhengzhou, China

**Keywords:** *Toxoplasma gondii*, seroprevalence, dogs, feces, PCR, China

## Abstract

**Background:**

*Toxoplasma gondii* can infect almost all warm-blooded animals, including humans and dogs. Humans can become infected with *T. gondii* by petting dogs that have eaten or contacted infected cat feces. The aim of this study was to evaluate *T. gondii* infections in dogs from central China. From 2015 to 2021, a total of 536 dog samples (195 fecal, 81 hearts, and 260 serum samples) from Henan Province were collected. Heart juice or serum samples (n = 341) were tested for *T. gondii* antibodies using the modified agglutination test (MAT). Fresh myocardium (n = 6) and blood (n = 2) samples were bioassayed in mice.

**Results:**

The present study showed that 4.40% (15/341) of the dogs were seropositive for *T. gondii* by MAT (cut-off, 1:25) and 4.10% (8/195) of dog feces contained *T. gondii* DNA. No *T. gondii* DNA was found in any myocardium (n = 81) or blood (n = 2) samples. The viable *T. gondii* strain was not isolated from any myocardium or blood samples (n = 8). Compared to the prevalence of *T. gondii* antibodies in dogs sampled from 2015 to 2018, the prevalence significantly declined from 2020 to 2021 (*P* < 0.05). Gender and age were not risk factors for dogs infected with *T. gondii* in this study. However, compared to other sources, dogs from Zhoukou City (close to the Yellow River) or from pet shops showed significantly higher prevalence for *T. gondii* (*P* < 0.05).

**Conclusion:**

A total of 4.29% dogs were infected by *T. gondii* (23/536, 8 of 195 fecal samples, 2 of 260 serum, and 13 of 81 heart juice samples). This is the first survey of *T. gondii* infection in dog feces from China. Dogs were exposed to *T. gondii*, and they could act as mechanical transmitters of *T. gondii*.

## Background

Toxoplasmosis is caused by an obligate, intracellular, *Toxoplasma gondii*. *T. gondii* can infect almost all warm-blooded animals, including humans and dogs. Although *T. gondii* infection appears asymptomatic in most species, there are severe risks for immunosuppressed individuals, pregnant women, sheep, new world monkeys, and marsupials, as it may cause severe health implications to fetuses or acute infection (Dubey, 2010; Shapiro et al., 2019; Calero-Bernal and Gennari, 2019; Dubey et al., 2020).

Recently, we reviewed *T. gondii* infection in dogs and found that *T. gondii* antibodies were found in dogs worldwide (Dubey et al., 2020). Many fatal cases of canine toxoplasmosis have been reported, where ulcerative dermatitis, rear limb paralysis, and myocarditis were the main clinical presentations (Dubey, 2010; Dubey et al., 2020; Dorsch et al., 2022). Humans can become infected with *T. gondii* by petting dogs that have eaten or have been in contact with infected cat feces. The oocysts of *T. gondii* ingested by dogs may pass through the digestive tract and remain infectious (Lindsay et al., 1997). Dogs can mechanically transmit *T. gondii* oocysts to humans through their body surfaces, mouth, and feet (Dubey et al., 2020). Therefore, *T. gondii* infection in dogs can be an indicator for the level of environmental contamination for humans (Meireles et al., 2004). Additionally, because dog meat serves as food for humans in some regions (Cui and Wang, 2001; Chevalier et al., 2021; Tasiame et al., 2021), consumption of undercooked dog meat containing *T. gondii* cysts also poses a health risk.

**Figure 1:**
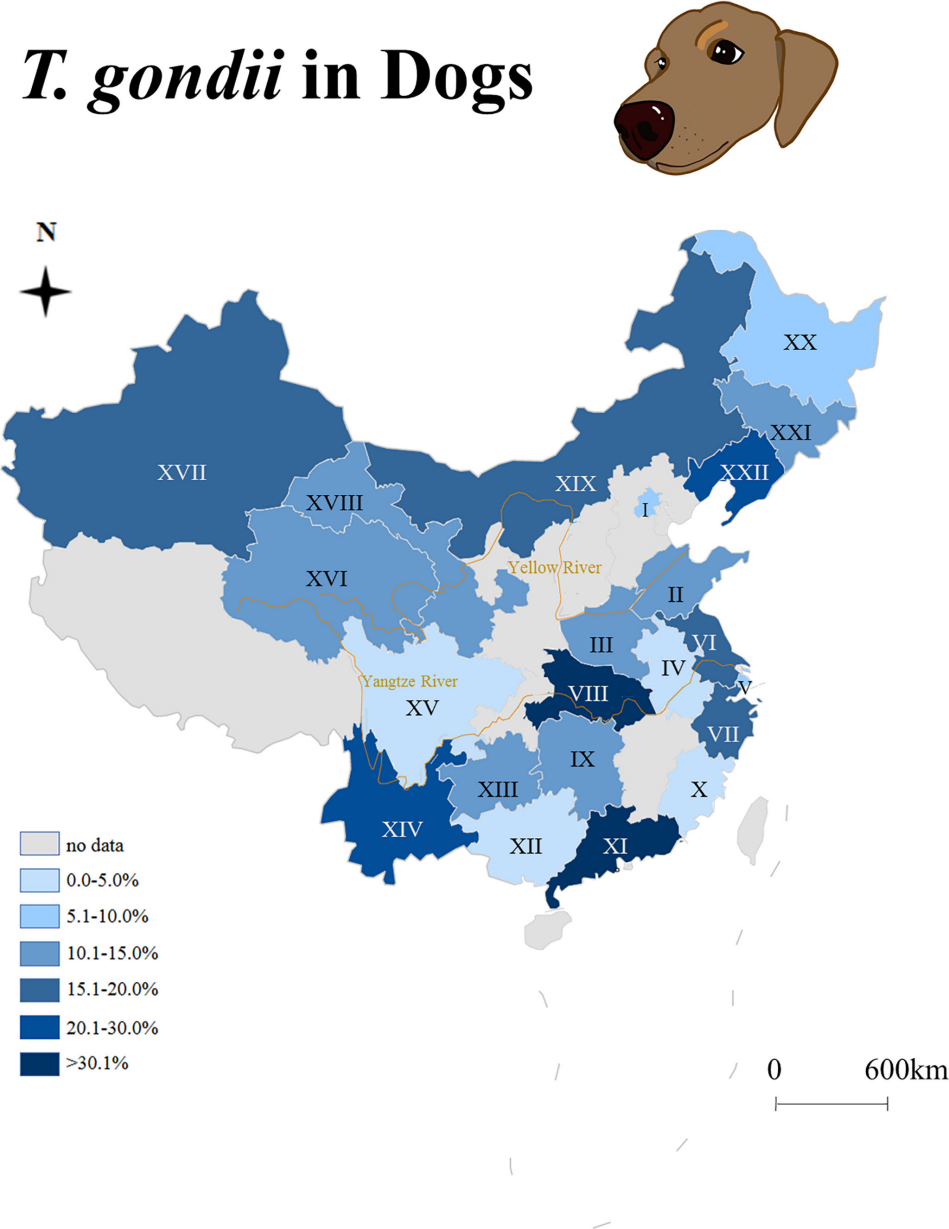
Seroepidemiology of *Toxoplasma gondii* in dogs from China (2009–2021). I: Beijing, II: Shandong, III: Henan, IV: Anhui, V: Shanghai, VI: Jiangsu, VII: Zhejiang, VIII: Hubei, IX: Hunan, X: Fujian, XI: Guangdong, XII: Guangxi, XIII: Guizhou, XIV: Yunnan, XV: Sichuan, XVI: Qinghai, XVII: Xinjiang, XVIII: Gansu, XIX: Inner Mongolia, XX: Heilongjiang, XXI: Jilin, XXII: Liaoning.

China has an estimated 27 million domestic dogs, ranking third worldwide, and has an unknown number of wild dogs. The seroepidemiology of *T. gondii* in dogs from China is summarized in **Figure 1**. For China, there are few reports on clinical toxoplasmosis in dogs, no viable *T. gondii* strain has been isolated, and little is known about the mechanical excretion of *T. gondii* oocysts from dog feces. The objective of the present study was to investigate the prevalence of *T. gondii* infections in dogs (bodily fluids and feces) in China, and an attempt was made to isolate viable *T. gondii* strains.

## Materials and Methods

### Collection of Dog Hearts, Blood, and Fecal Samples

From 2015 to 2021, 81 fresh dog hearts were collected from slaughterhouses, 260 dog blood samples were collected from pet hospitals, and 195 dog fecal samples were collected from farms, shelters, pet shops, police dog-breeding bases, and hospitals (Henan Province, China) (**Tables 1**, **2**). The fecal samples were obtained with the help of the dog owners, and the feces (5 g) were collected from rectum and placed in plastic bags. Henan Province (34.90°N, 113.50°E) is located in a warm temperate zone and in the trans-subtropical southern region. In the past 10 years, the annual temperature has ranged from 12.9°C to 16.5°C, and the annual precipitation has ranged from 464.2 to 1,193.2 mm.

**Table 1:** Serological investigation of *Toxoplasma gondii* infection in dogs from Henan Province, China.

City	Samples received date	Age (years)			No. of samples	Positive no. in different titers by MAT									% (Positive no./test no.)
		≤1	>1	NK *^a^*		1:5	1:10	1:25	1:50	1:100	1:200	1:400	1:800	1:1600	
Kaifeng	April 29, 2015	NK	NK	27	27 hearts *^c^*	-*^b^*	–	1 *^d^*	–	–	0	–	–	–	3.70 (1/27)
Zhoukou	May 10, 2015	18	15	0	33 hearts *^c^*	–	–	1	0	7 *^d^*	2 *^d^*	0	0	0	22.22 (12/54)
	May 11, 2015	11	10	0	21 hearts *^c^*	–	–	2 *^d^*	0	–	0	0	0	0
Zhengzhou	June 28, 2018	4	9	0	13 sera	–	–	0	–	–	0	–	–	–	0.79 (2/254)
	December 13, 2018	0	1	0	1 blood	–	–	0	–	–	0	1 *^e^*	0	0
	October 12, 2020	24	57	2	83 sera	0	0	–	–	–	–	–	–	–
	November to December 2020	15	36	2	53 sera	0	0	–	–	–	–	–	–	–
	January 16, 2021	1	0	0	1 blood	0	1 *^e^*	0	0	0	0	–	–	–
	January 19, 2021	4	15	1	20 sera	0	0	–	–	–	–	–	–	–
	January 2021	7	76	0	83 sera	0	3	0	0	0	1	–	–	–
Puyang	October 07, 2020	0	6	0	6 sera	0	0	–	–	–	–	–	–	–	0 (0/6)
Total					81 hearts 260 sera or blood 341 samples										16.05 (13/81) 0.77 (2/260) 4.40 (15/341)

a NK: Unknown.

b Not tested.

c One heart juice sample from each heart.

d Myocardium samples biossayed in mice.

e Dog blood samples bioassayed in mice, collected at pet hospitals.

**Table 2:** Prevalence and risk factors of *Toxoplasma gondii* in dog feces tested by PCR.

Variable		No. of samples	No. of positive	Prevalence % (95% CI)	OR (95% CI)	*P-value*
Location	Zhengzhou	117	6	5.13 (2.14–10.97)	1.351 (0.3251–6.761)	0.7172
	Puyang	52	2	3.85 (0.32–13.72)	1	
	Kaifeng	20	0	–	–	
Age (years)	≤1	34	6	17.65 (7.97–33.89)	–	–
	>1	40	0	–	–	
Gender	Male	33	4	12.12 (4.21–27.93)	1.448 (0.3107–8.113)	0.6835
	Female	23	2	8.70 (1.25–27.97)	1	
Area	Urban	117	6	5.13 (2.14–10.97)	1.769 (0.4255–8.794)	0.4867
	Rural	71	2	2.82 (0.19–10.29)	1	
Breed	Cross-breed	23	2	8.70 (1.25–27.97)	1.619 (0.2903–7.327)	0.5899
	Pure breed	72	4	5.56 (1.77–13.84)	1	
Source	Pet shops	19	4	21.05 (7.95–43.89)	7.467 (1.559–40.73)	0.0130*
	Countrysides	58	2	3.45 (0.27–12.41)	1.143 (0.1744–7.472)	0.8954
	Shelters	66	2	3.03 (0.22–11.01)	1	
	Hospitals	5	0	–	–	
	Dog farms	26	0	–	–	
	Police dog bases	21	0	–	–	
Total		195	8	4.10 (1.96–8.02)		

*Statistically significant (P < 0.05).

OR, odds ratio; CI, confidence interval.

Heart juice samples from dog myocardium were centrifuged for 10 min at 2,000 rpm; serum samples were subsequently separated. Heart juice, serum, and myocardium samples were stored at 4°C and tested for *T. gondii* antibodies or bioassayed in mice within 2 days. Fecal samples were cultured at room temperature for 2 weeks so that oocysts could sporulated.

### Detection of *T. gondii* Antibodies in Dog Heart Juice and Sera

Sera (n = 260) and heart juice (n = 81) from 341 dogs were tested for *T. gondii* antibodies using a modified agglutination test (MAT) (Dubey and Desmonts, 1987). Whole formalin-treated *T. gondii* tachyzoites were obtained from the University of Tennessee Research Foundation (Dr. CL Su, Knoxville, TN, USA). All heart juice and serum samples collected from 2015 to 2018 were tested at a titer of 1:25; the dilution was subsequently doubled to a titer of 1:1,600 (**Table 1**). Serum samples collected from 2020 to 2021 were tested at a titer of 1:5 and 1:10. Positive samples were tested at a titer of 1:25; the dilution was subsequently doubled to a titer of 1:200 (**Table 1**). Negative and positive controls were included in each plate. Heart juice and serum samples with a titer of ≥1:25 were considered as a sign of exposure to this parasite.

### Isolation of Viable *T. gondii* From Dog Myocardium and Blood Samples Through Mouse Bioassays

Myocardium (n = 6) and blood (n = 2) samples from dogs were bioassayed in mice following previously described methods (Dubey, 2010). Briefly, myocardia (50 g) were digested in a pepsin solution and inoculated in Swiss mice (n = 2–3) subcutaneously. Blood samples were centrifuged for 10 min at 2,000 rpm; the sediment was suspended in saline and injected into Swiss mice (n = 2) or gamma interferon (IFN-γ) knockout mice (n = 1) subcutaneously. Swiss mice were supplied by the Zhengzhou University Laboratory Animal Center (China). IFN-γ^-/-^ mice were purchased from the Jackson Laboratory (Stock No.: 002287; Bar Harbor, ME, USA).

Clinical symptoms in mice were recorded daily. The room temperature in the facilities was 22°C–24°C, and the humidity was 60%–70%. *T. gondii* tachyzoites or cysts in the lungs or brains of dead or euthanized mice were examined. If cysts or tachyzoites were not found in mouse tissues, the homogenized lung, brain, and myocardium were subcutaneously subpassaged into a new group of mice. Sera were collected from surviving mice at 30 days post-inoculation (DPI). Mouse sera at dilutions of 1:25 and 1:200 were tested for *T. gondii* antibodies by MAT.

### Detection of *T. gondii* DNA in Fecal, Myocardium, and Blood Samples

Oocysts were purified from dog fecal samples by the conventional sucrose flotation method at room temperature (Dubey, 2010). Floated materials were transferred to slides and checked by light microscopy. Parasite oocysts and eggs were differentiated based on their morphological characteristics. Oocysts were stored at −20°C in a refrigerator for further analysis.

DNA was extracted from the myocardium (n = 81), pepsin-digested myocardium juice (n = 6), blood (n = 2), and fecal flotation (n = 195) samples using a DNA extraction kit (DP304; Tiangen Biotech Co., Beijing, China). *T. gondii* DNA was amplified by polymerase chain reaction (PCR) which targeted the 529-bp repetitive DNA fragment of *T. gondii* (primer pair, Tox5–Tox8) (Reischl et al., 2003; Schares et al., 2008). The length of the PCR products was estimated to be 450 bp and included negative and positive controls.

### Statistical Analysis

Statistical analyses were performed using GraphPad Prism version 8.4.3 software (GraphPad Software Inc., San Diego, CA, USA). The results were analyzed by the chi-square or Fisher’s exact test. The Monte Carlo test of simulated data was implemented to assess the risk factors associated with *T. gondii* infection. A *P*-value of <0.05 was considered statistically significant.

## Results

### Serologic Investigation of *T. gondii* Infection in Dogs

In the present study, the MAT results indicated that 4.40% (15/341, 95% CI, 2.63–7.19) of the examined dogs were seropositive with titers of 1:25 in four dogs, 1:100 in seven, 1:200 in three, and 1:400 in one (**Table 1**). They were 13 of 81 hearts collected at slaughterhouses, and 2 of 260 blood samples from pet hospitals (**Tables 1**, **3**). The seroprevalence (sera, heart juice) of *T. gondii* infection in dogs from four cities ranged from 0% to 22%. The seroprevalence rates of *T. gondii* varied by region (**Table 3**). A significantly higher *T. gondii* seroprevalence was observed in Zhoukou when compared to other regions (*P* = 0.0323); there were no seropositive serum samples from Puyang.

**Table 3:** Seroprevalence and risk factors of *Toxoplasma gondii* infection in dogs tested by a modified agglutination test (MAT).

Factor	Classification standards	No. of samples	Total positive no.	Seroprevalence %	OR (95% CI)	*P-value*
Year	2015	81	13	16.05	2.485 (0.3625–28.36) *^a^*, 19.88 (3.021–213.9) *^b^*	0.3853 *^a^* 0.0001 *^b^* *
	2018	14	1	7.14	7.923 (0.3888–151.4) *^c^*	0.0925 *^c^*
	2020	142	0	–	–	
	2021	104	1	0.96	1	
Age (years)	≤1	84	5	5.95	1.519 (0.5550–4.503)	0.4628
	>1	225	9	4.00	1	
Gender	Female	142	8	5.63	1.026 (0.9741–1.088)	0.3109
	Male	155	5	3.23	1	
Location	Zhoukou	54	12	22.22	7.429 (1.163–82.39)	0.0323^*^
	Kaifeng	27	1	3.70	4.846 (0.3228–42.34)	0.1610
	Zhengzhou	254	2	0.78	1	
Source	Slaughterhouse	81	13	16.05	24.66 (6.153–110.8)	<0.0001*
	Pet hospitals	260	2	0.77	1	

*Statistically significant (P < 0.05).

a 2015 vs. 2018.

b 2015 vs. 2021.

c 2018 vs. 2021.

OR, odds ratio; CI, confidence interval.

The prevalence of *T. gondii* antibodies in dogs sampled from central China significantly declined from 2015 to 2021 (*P* = 0.0001) with positive serum rates of 16.05% (13/81, 95% CI, 9.49–25.69) in 2015, 7.14% (1/14, 95% CI, 0.01–33.54) in 2018, and 0.96% (1/104, 95% CI, 0.01–5.77) in 2021; there were no positive serum samples in 2020 (0/142). Regarding gender and age, females had a higher seropositive rate than males, and dogs that were ≤1-year-old had a higher seropositive rate than dogs that were >1-year-old; however, no significant differences were detected (**Table 3**).

### Microscopic Examination and Molecular Assays of *T. gondii* in Fecal Samples

In this survey, dogs were frequently mix-infected with 2–3 parasite species, including roundworm, isospora, whipworm, tapeworm, and hookworm by fecal microscopic examination. No *T. gondii*-like oocysts were found under light microscopy. Samples were checked for *T. gondii* nucleic acid by PCR. *T. gondii* DNA were amplified from 4.10% (8/195) dog fecal samples using the primer pair, Tox5–Tox8 (**Table 2**).

A significantly higher prevalence of *T. gondii* in fecal samples was observed in ≤1-year-old dogs when compared to dogs that were >1-year-old (*P* < 0.05). A significantly higher prevalence of *T. gondii* was observed in dogs from pet shops when compared to other sources (*P* = 0.0130). Fecal samples from males, urban regions, and crossbred dogs had a higher prevalence of *T. gondii*; however, no significant differences were observed (**Table 2**).

### *T. gondii* Isolation and DNA Detection

In this study, six fresh myocardium (seropositive for *T. gondii*) and two blood (one at 1:400, one at 1:10) samples were bioassayed in mice (**Table 4**). Unfortunately, the viable *T. gondii* strain was not successfully isolated from any tissues or blood samples. After mice were bioassayed, only one group of mouse sera (dog blood: ID# December 13, 2018; two positive mice of three inoculated mice) showed seroconversion for *T. gondii* at 39 DPI. However, after subpassage, none of the mice were positive for *T. gondii* infection by serology or etiology. None of the myocardium (n = 81), myocardium digestion fluid (n = 6), or blood (n = 2) samples contained *T. gondii* DNA.

**Table 4:** Isolation of *T. gondii* from dogs by bioassay in mice.

Sample ID	MAT titers	PCR TOX5/8	No. positive/no. inoculated	Mice *T. gondii* antibody in different titers by MAT		Passaged groups results *^c^*
				1:25	1:200	
20150430#D18 heart	1:25	Heart digested liquid -	0/2 *^b^*	- *^a^*	–	0/1
20150510#D38 heart	1:200	Heart digested liquid -	0/3	–	–	0/1
20150510#D53 heart	1:100	Heart digested liquid -	0/3	–	–	0/1
20150510#D55 heart	1:200	Heart digested liquid -	0/3	–	–	0/1
20150511#D12 heart	1:25	Heart digested liquid -	0/2	–	–	0/1
20150511#D16 heart	1:25	Heart digested liquid -	0/2	–	–	0/1
20181213 Dog blood	1:400	–	2/3	+ *^d^*	+	1/2
20210116 Dog blood	1:10	–	0/3	–	–	0/1

a Negative.

b No. of positive mice/no. of inoculated mice.

c No. of positive groups/no. of passaged groups.

d Positive.

## Discussion

Dogs are susceptible to *T. gondii* with a seroprevalence of 1.9% to 97.0% in the world (Dubey et al., 2020). Dogs have a keen sense of smell and the behavior of rolling in feces, thereby increasing their exposure to *T. gondii* (Frenkel et al., 2003; Dubey et al., 2020). Canine toxoplasmosis can also be caused by ingesting oocysts or meat containing *T. gondii* cysts (Yan et al., 2012; Calero-Bernal and Gennari, 2019). A previous epidemiological study detected a uniform prevalence of *T. gondii* between humans and dogs, which may be because they lived in the same environment, indicating that dogs may be a good sentinel species of *T. gondii* exposure for humans (Tenter et al., 2000; Meireles et al., 2004).

The seropositive rate was 4.40% (15/341) of the dogs by MAT. They were 13 of 81 hearts collected at slaughterhouse, and 2 of 260 blood samples collected from pet hospitals (**Table 1**). Hearts were collected in 2015, while blood samples were collected mainly in 2020–2021. Compared to the seroprevalence of *T. gondii* antibodies in dogs sampled from 2015 to 2018 in Henan Province, the prevalence significantly declined from 2020 to 2021. Previous studies have reported a *T. gondii* prevalence of 34.9% (83/238), 20.8% (26/125), and 3.23% (1/31) in dogs sampled from Henan Province (Wang et al., 2012; Yang et al., 2014; Qian et al., 2015). Recently, China has seen a rapid development of its economic and health industries. The increased economic and cleanliness levels may play a role in suppressing *T. gondii* transmission and distribution in the environment.

In the present study, geographic location was a risk factor associated with *T. gondii* seroprevalence in dogs. Compared to other regions, the *T. gondii* prevalence in dogs from Zhoukou was significantly higher. This may be due to the following: (1) Henan Province is located downstream of the Yellow River. Zhoukou is closer to the Yellow River than other cities, and *T. gondii* oocysts may be transported *via* freshwater, thereby posing a threat to animals residing close to the river. (2) Samples from Zhoukou were collected in 2015, which is relatively earlier than in cities of Zhengzhou and Puyang. Regarding *T. gondii* infection in this study, gender and age were not risk factors for dogs, and the lack of relationship with gender was in agreement with the findings of other studies (Lopes et al., 2011; Yang et al., 2013; Nguyen et al., 2020). However, a previous study found that seroprevalence in dogs increased with age (Dubey et al., 2020), which may be related to the low seroprevalence of *T. gondii* in dogs or small sample size in this study.

Environmental pollution due to dog feces is a public health concern and constitutes a health hazard to humans (Ainmode et al., 2016). When dogs ingest cat feces containing *T. gondii* oocysts, oocysts remain viable after passing through the digestive tract (Lindsay et al., 1997). Here, *T. gondii* DNA was detected in the feces of 8/195 dogs sampled from Henan Province. The seroprevalence levels of *T. gondii* in domestic cats in Henan Province were 50% (21/42) (Yang et al., 2015), 21% (178/843) (Wang et al., 2017), and 7% (2/28) (Yang et al., 2017). These results indicated that the environment may be polluted by *T. gondii* oocysts. Humid tropical climates are conducive for maintaining oocysts in the soil and water, thereby contributing to its widespread dissemination (Afonso et al., 2006). *T. gondii* infection in dog feces is rarely reported on a global scale. In a previous study, *T. gondii* DNA was detected in the feces of 4/120 dogs sampled from the United States (Munoz and Mayer, 2016). *T. gondii* isolates (TG-dgGER1, TG-dgGER2) were successfully isolated from 2/24,089 fecal samples (Schares et al., 2005).

Compared to older dogs, there was a significantly higher prevalence of *T. gondii* DNA in feces from ≤1-year-old dogs. This may be explained by the fact that puppies are more active than adults. Dogs from pet shops had a higher prevalence of *T. gondii* DNA in their feces than those from shelters, hospitals, or farms. Pet shops often sell many kinds of animals, including cats, dogs, birds, and fish, indicating that the pet shop environment may increase the exposure of dogs to *T. gondii* oocysts. Gender, breed, and source area were not risk factors associated with *T. gondii*-positive rates in dog fecal samples based on the *T. gondii* DNA results in this study. In order to investigate *T. gondii* infection in dogs, excluding serological tests, fecal samples may be an excellent option for etiology detection in the future. Moreover, feces are easier to obtain, especially for vigilant and fierce stray dogs or wild canines. Bioassays of dog feces *via* cats or mice could further verify *T. gondii* infectivity.

In our previous study, *T. gondii* was not isolated from the myocardium of 14 seropositive dogs (MAT ≥ 10) sampled from 2013 to 2014 (Yang et al., 2014). Unfortunately, in this study, *T. gondii* was not isolated from dog myocardium (n = 6, MAT ≥ 25) or blood (n = 2, MAT ≥ 10) samples. This may be due to the following: (1) the low virulence of *T. gondii* in dogs or (2) the low density of parasite loading in dog tissues. The fact that no *T. gondii* DNA was found in the myocardium (n = 81), myocardium digestion fluids (n = 6), or blood (n = 2) also confirms this hypothesis. Although serological results indicated contact of dogs with *T. gondii*, tissues were negative for the presence of the parasite. *T. gondii* infection might occur when consuming dog meat. The lack of detection of the parasite by molecular assays or biological assays in chronic *T. gondii* infection might be due to the limited sample size analyzed (0.5 g tissue or 50 g tissue), the uneven distribution of tissue cysts, and perhaps the low number of *T. gondii* tissue cysts in the tissues.

## Conclusions

This is the first survey of *T. gondii* infection from dog fecal samples in China. The results showed that 4.40% (15/341) of the dogs were seropositive by MAT and 4.10% (8/195) dog feces contained *T. gondii* DNA. Dogs were exposed to *T. gondii*; they could act as mechanical transmitters of *T. gondii*.

## Data Availability Statement

The raw data supporting the conclusions of this article will be made available by the authors, without undue reservation.

## Ethics Statement

The animal study was reviewed and approved by Beijing Association for Science and Technology (SYXK [Beijing] 2007-0023).

## Author Contributions

NZ performed the laboratory tests and data analysis and wrote the manuscript. LY, SX, and WH participated in the sample collection and laboratory testing. YJ and YY designed the study protocol, analyzed the results, and wrote the manuscript. All authors contributed to the article and approved the submitted version.

## Funding

This study was financed by the Natural Science Foundation of Henan Province, China (202300410214), and the Key Research Projects of Henan Higher Education Institutions (21A230009).

## Conflict of Interest

The authors declare that the research was conducted in the absence of any commercial or financial relationships that could be construed as a potential conflict of interest.

## Publisher’s Note

All claims expressed in this article are solely those of the authors and do not necessarily represent those of their affiliated organizations, or those of the publisher, the editors and the reviewers. Any product that may be evaluated in this article, or claim that may be made by its manufacturer, is not guaranteed or endorsed by the publisher.

## Abbreviations

MAT, modified agglutination test; PCR, polymerase chain reaction.
